# Evaluating resilience levels and their association with spiritual health and other factors among university students from South Korea and Japan; a comparative cross-sectional study

**DOI:** 10.1186/s12889-025-24738-1

**Published:** 2025-10-14

**Authors:** Hira Taimur, Ishtiaq Ahmad, Yoshihisa Shirayama, Miyoko Okamoto, Ji Eon Kim, Eun Woo Nam, Motoyuki Yuasa

**Affiliations:** 1 Department of Global Health Research, Graduate School of Medicine, Juntendo University, Tokyo, Japan; 2 Faculty of International Liberal Arts, Juntendo University, Tokyo, Japan; 3 Department of Health and Medical Welfare, Pai Chai University, Daejeon, Republic of Korea; 4 Department of Health Administration, Yonsei University Graduate School, Wonju, Republic of Korea; 5 Yonsei Global Health Centre, Yonsei University, Wonju, Republic of Korea

**Keywords:** 14-item resilience scale, Japan, Purpose in life scale, Resilience, South Korea, Spiritual health, University students

## Abstract

**Background:**

Given the high prevalence of mental health problems among university students in South Korea and Japan, understanding and fostering their inner strengths is important. This study assessed resilience and its correlates among undergraduate students from both countries.

**Methods:**

A cross-sectional survey was conducted in Wonju City (South Korea) and Bunkyo City (Japan) using an online questionnaire. The survey included validated instruments: the 14-item Resilience Scale and the 20-item Purpose in Life (PIL) Scale. A total of 598 valid responses were included in the analysis. Descriptive statistics were used to assess levels of resilience and spiritual health, while quantile regression was used to examine associations between resilience and spiritual health, as well as socio-demographic and lifestyle factors.

**Results:**

Low resilience was observed in 57.1% of South Korean students (median age 21; 71.5% female) and 67.3% of Japanese students (median age 19; 58% female). Purpose in life (PIL) was positively associated with resilience across all quantiles in both groups (Korea β = 0.53–0.62; Japan β = 0.56–0.65). Among Korean respondents, club affiliation, age, and female gender were linked to higher resilience at specific quantiles. Among Japanese respondents, financial satisfaction was inversely associated with resilience at the 25th and 50th quantiles (β = − 2.52 to − 4.76), while academic satisfaction was positively associated at the 25th quantile (β = 4.19).

**Conclusion:**

Given the high prevalence of low resilience, targeted strategies to enhance resilience are needed. The findings highlight purpose in life as a key positive correlate, alongside sociodemographic and financial factors with variable effects across resilience levels.

**Supplementary Information:**

The online version contains supplementary material available at 10.1186/s12889-025-24738-1.

## Background

The present era is witnessing increased complexities of contemporary life due to global connectedness, technological innovations, and evolving cultural standards. Simultaneously, there has been an observable increase in both natural and technological disasters, as well as political conflicts. Mental health assessment is paramount in this context. Since 1990, mental disorders have Jumped up in ranking of top causes of health loss worldwide from 12th to 7th position, with depressive and anxiety disorders being the highest in 20 to 35 years age group in 2020 [1]. Depression was projected to be one of the three leading causes of burden of disease (along with HIV/AIDS and heart disease) until 2030 [2]. Recent evidence suggests that the mean age of onset of most mental health disorders is around 21 years [3]. A higher prevalence of depressive disorder and anxiety has been reported among university students as compared to the general population, despite being a socially advantaged group [4, 5]. Mental health issues are a rising public health concern in East Asian countries of South Korea and Japan. Recent studies documented a depression prevalence of 22.4% and 29.9% among South Korean and Japanese university students, respectively [6, 7], along with a widespread social stigma attached to receiving professional psychological help [8]. Furthermore, among OECD countries, South Korea and Japan had the highest and third-highest suicide rates in 2021 respectively [9], with suicide being the leading cause of death among university students in both countries [10, 11]. Despite much effort, the prevalence of mental illness remains high among the youth of both countries [12, 13]. An important route to lighten the burden of mental health problems and psychological distress is to enhance the ability to deal with stressors effectively.

Resilience refers to the defense mechanism that enables people to maintain their mental health despite psychological and physical adversity. This concept is in line with the Salutogenic Model of Health Promotion by Antonovsky [14], which focuses on determinants of health rather than disease. The resilience research aims to focus on protective mechanisms that protect people from developing mental disorders, thereby shifting the focus from mitigating external risks to nurturing internal strengths and capacities. Literature has reported different definitions of resilience, all of which conceptualize resilience as a dynamic construct that enables the individual to manage stress and return to a normal state [15, 16]. The resources within the individual, their environment, and interaction between the two facilitate this adapting and bouncing-back capacity in the face of adversity [17]. There is ample evidence in the literature that shows a positive association of resilience with well-being and a negative association with mental health problems [18, 19]. Resilience is considered modifiable and can be improved by interventions [20, 21]. Varying levels of resilience are found in the literature among students, along with different correlating factors, with most published studies reporting moderate levels of resilience among university students [22–24]. Despite its importance and its correlation with stress and mental health, resilience is understudied among university students in South Korea and Japan. Given the considerable burden of mental health issues such as depression in students from both these countries, there is a need for conducting resilience studies in the South Korean and Japanese contexts.

One potential correlate that we explored in this study is spiritual health. Spiritual health is considered the fourth dimension of health. This area is rather ambiguous with no consensus in terms of definition or measurement. One reason behind this ambiguity is the interchangeable use of the terms spirituality and religiosity in many cultures. The authors of this study have discerned “spiritual health” as what gives purpose in life, as opposed to “religion” which is an organized system designating beliefs, rituals, practices, and behaviors that enhance the relationship with a higher power [25]. While religious affiliation is a choice, spirituality is a need. The purpose in life is a core implication of spiritual health common to all definitions [26]. Spiritual health is emerging as an important notion of positive psychology since researchers have suggested it as a possible predictor of psychological well-being [27], and a protective factor against many mental health disorders such as depression, anxiety, and suicidal behavior [28]. Despite increasing interest in spiritual health as an important dimension of health, international research based in the peer-reviewed domain is limited. While researchers have studied the correlation of resilience with constructs such as the sense of coherence, self-transcendence, social support, and stress inventory, the association of resilience and spiritual health is understudied and requires further exploration.

The present study has the following objectives: [1] to assess the levels of resilience and spiritual health among university students in South Korea and Japan; [2] to explore the relationship between spiritual health and resilience within these populations; and [3] to identify the sociodemographic and lifestyle factors associated with resilience among university students in both countries. A significant association of the aforementioned factors with resilience is hypothesized. Identifying the correlates and including them in intervention programs has possible benefits in improving the holistic health of this population. Understanding these relationships may inform future intervention strategies and early identification efforts in university mental health settings.

## Methods

### Study design

This is a web-based international comparative cross-sectional study aimed at assessing the level of resilience among university students in Japan and South Korea and investigating the association between resilience and spiritual health as well as other factors. Online survey methodology was employed due to its advantageous features, including lower costs, reduced implementation time, high response rate, and good internet coverage on university campuses.

### Study population and data collection

Using a 10% difference in effect size, α error probability of 0.05 (two-sided), and a power (1-β) of 0.90, the sample size calculated by 1-sample estimation of proportion in Stata 18.0 was 257. After a 10% inflation to compensate for possible non-response bias, the final sample size was set at 290 for each country [29]. The survey periods were April 1 to 30, 2024, in South Korea, and May 15 to June 15, 2024, in Japan. The time period of survey was carefully selected to avoid any stresses related to examinations. The survey was conducted at three universities in Wonju, South Korea, and one university in Tokyo, Japan, selected through convenience sampling. Participants were recruited by invitation through verbal announcements in classes and seminars to complete the form during the scheduled survey period. This recruitment method was selected due to its practicality in reaching a broad range of students across various faculties during class hours, given institutional constraints on direct student contact and the need for efficient, ethically approved access to participants. Inclusion criteria are as follows: nationals of South Korea and Japan (including dual nationality holders), 18 to 26 years old, currently enrolled in a bachelor’s degree program.

We created online questionnaires using Google Forms in both Korean and Japanese languages. The survey was conducted by sharing the Uniform Resource Locator (URL) with students who agreed to participate in the survey. Before filling out the form, the participants were informed about the study objectives, data storage methods, conflicts of interest, and tentative time to fill out the form (15 min approx.). A written informed consent was obtained from all participants. Responses were eliminated if they had missing values or did not meet the inclusion criteria. We received 300 responses from South Korea and 336 responses from Japan (totaling 636). After excluding participants who did not fit the inclusion criteria (*n* = 35) and missing data (*n* = 3), our study retained 298 (99.3%) responses from South Korea and 300 (89.3%) responses from Japan. The number of responses from each country met the minimum sample size requirement for data analysis.

### Study instruments

The dependent variable in our study is resilience which was measured using Wagnild’s 14-item Resilience Scale instrument (RS14). It is a concise form of The Resilience Scale (Wagnild & Young, published 1993), the first instrument to measure resilience directly. Each of the 14 items is responded on a 7-point Likert scale. The resilience variable is constructed by summing up the scores for each item, with a total score ranging from 14 to 98. Higher scores indicate superior levels of resilience. We used authentic Japanese and Korean translations of the scale for which reliability and validity studies have already been conducted [30, 31]. We used the cutoff values as follows: “low resilience” at scores of 14 to 73, “moderate resilience” at scores of 74 to 81, and “high resilience” at scores of 82 to 98. These cutoffs are based on the scoring guidelines established by the original scale developers, derived through psychometric validation in diverse populations. Cronbach alpha for RS14 in our study was 0.91.

The independent variable in our study is spiritual health measured by the Purpose in Life questionnaire (PIL). We consider this instrument appropriate to assess spiritual health in our study population (which is mostly atheist), as most other validated instruments included items related to God or religion, making them inappropriate to use in this population. This scale was developed in 1964 by Crumbaugh and Maholick based on Viktor Frankl’s concept of the existential vacuum. It is a 20-item instrument, responses for each item are recorded on a 7-point Likert scale. The variable is constructed by summing up the scores on each item, giving a total score ranging from 20 to 140 [32]. The original PIL test was written in English; and since then, it has been translated into many languages. We included authentic Japanese and Korean translations of this instrument in our questionnaire, as used by some previous studies [33–35]. We used the cutoff values suggested by test manual authors as follows: lack of clear meaning and purpose indicated by scores between scores 20 and 91 as “low sense of purpose” and indecisive range between scores 92 and 112 as “moderate sense of purpose” and definite purpose indicated by scores 113 and above as “high sense of purpose” [32]. Cronbach’s alpha for PIL in our study was 0.91.

Data on other independent variables such as sociodemographic and lifestyle factors were collected using following variables: age, gender, religious affiliation, self-reported knowledge of their own history and culture, academic major, self-reported academic satisfaction, affiliation with club/society, living arrangement, self-reported health status, self-reported financial satisfaction, body mass index (BMI), self-reported sleep quality (in last 4 weeks), self-reported physical activity (in last 7 days) and having any volunteering experience. The question phrasing and response categories are given in supplementary file 1. The demographic questions were initially generated in English and later translated and back-translated by local researchers into the respective languages (Korean and Japanese).

### Statistical analysis

Descriptive statistics summarized respondent characteristics using frequencies and percentages for categorical variables and medians with interquartile ranges for continuous variables. The Shapiro–Wilk and Breusch–Pagan tests were used to assess normality and heteroskedasticity in the data, respectively. Group differences were evaluated using Mann–Whitney U tests for continuous variables and Chi-square or Fisher’s exact tests for categorical variables as appropriate. Although resilience and purpose in life scores were categorized for descriptive purposes, they were treated as continuous variables in all inferential analyses.

Univariate quantile regression analyses were conducted to identify unadjusted associations between resilience and various independent variables. Subsequently, multivariate quantile regression models were fitted separately for Korean and Japanese respondents to assess adjusted associations, allowing for population-specific effects and cultural differences to be accounted for, as well as to facilitate direct comparison between the two groups. Multicollinearity was evaluated using variance inflation factors (VIF), with values < 5 indicating no significant multicollinearity. Quantile regression was selected for its robustness to non-normal distribution and heteroskedasticity, and its ability to estimate associations across the distribution of dependent variable. Models were estimated at the 25th, 50th (median), and 75th quantiles to capture effects at different resilience levels.

Statistical significance was set at *p* < 0.05, and 95% confidence intervals were reported. All analyses were performed using Stata version 18.0 (StataCorp, College Station, TX, USA).

### Ethical consideration

This study was conducted in accordance with the ethical principles outlined in the Declaration of Helsinki. The study was approved for conduct online, targeting both South Korean and Japanese students by the Ethical Review Board of Juntendo University, Tokyo, Japan (authorization number E23-0363). Written informed consent was obtained from all participants prior to data collection. Participants were clearly informed of their right to refuse participation on the first page of the online survey. To ensure privacy, all responses were anonymized, and no personally identifiable information was collected. Data were securely stored in password-protected files, with access restricted exclusively to the research team. Due to anonymous nature of the survey, participants were informed that withdrawal of their data after submission was not possible.

## Results

Data from 598 participants (298 from South Korea and 300 from Japan) was analyzed. The median age of South Korean participants was 21 years. The majority were females (71.5%), had no religious affiliation (64.4%), were living with families (68.1%), involved in volunteer activities (90.9%), affiliated with clubs/societies (53.4%) and had normal BMI (59.3%). A good proportion of the South Korean participants had moderate knowledge of their history and culture (42.0%), were self-reportedly satisfied academically (53.7%) and financially (41.9%); had good self-reported health status (53.7%) and good sleep quality (38.3%).

The median age of Japanese students was 19 years. The majority were females (58.0%), had no religious affiliation (85.0%), were living with families (76.0%), not involved in volunteer activities (63.0%), not affiliated with activity clubs (63.3%), and had normal BMI (74.0%). Most of the Japanese participants had low knowledge of their own history and culture (68.3%) and were academically satisfied (50.3%) but financially neither satisfied nor dissatisfied (51.0%). Also, majority self-reported good health status (51.3%) but neither good nor bad sleep quality (40.3%). Low physical activity was self-reported by all study participants.

There were significant differences between South Korean and Japanese students in terms of gender, age, religion, knowledge of heritage, BMI, affiliation with clubs/society, volunteer activities, living arrangements, self-reported financial satisfaction, self-reported sleep quality, and level of resilience. No significant differences were found between the two study populations in terms of self-reported physical activity, self-reported academic satisfaction, self-reported health status, and spiritual health (Table 1).

**Table 1:** Respondent demographic characteristics by country

	South Korea *n* = 298	Japan *n* = 300	*p*-value
Gender			
Males	85(28.5%)	124(41.3%)	0.001
Female	213(71.5%)	174(58.0%)	
Others	0(0.0%)	2(0.7%)	
Age median (IQR)	21(20–23)	19(18–20)	< 0.001
Religion			
No religion	192(64.4%)	255(85.0%)	< 0.001
Buddhists	16(5.4%)	29(9.7%)	
Christians	82(27.5%)	6(2.0%)	
Shintos	8(2.7%)	9(3.0%)	
Knowledge of own history and culture			
Low	93(31.2%)	205(68.3%)	< 0.001
Moderate	125(41.9%)	77(25.7%)	
High	80(26.9%)	18(6.0%)	
Physical activity			
None	155(52.0%)	176(58.7%)	0.261
1–2 times	110(36.9%)	96(32.0%)	
> 3 times	33(11.1%)	28(9.3%)	
BMI			
Skinny	71(23.9%)	62(20.7%)	< 0.001
Normal	176(59.3%)	222(74.0%)	
Overweight	39(13.1%)	14(4.7%)	
Obese	11(3.7%)	2(0.7%)	
Students affiliated with club/society			
No	139(46.6%)	190(63.3%)	< 0.001
Yes	159(53.4%)	110(36.7%)	
Volunteers			
No	27(9.1%)	189(63.0%)	< 0.001
Yes	271(90.9%)	111(37.0%)	
Living arrangement			
Living alone	45(15.1%)	69(23.0%)	< 0.001
With family	203(68.1%)	228(76.0%)	
Dormitory	50(16.8%)	3(1.0%)	
Academic satisfaction			
Mostly dissatisfied	32(10.7%)	40(13.3%)	0.553
Neither satisfied nor dissatisfied	106(35.6%)	109(36.3%)	
Mostly satisfied	160(53.7%)	151(50.3%)	
Financial satisfaction			
Mostly dissatisfied	53(17.8%)	87(29.0%)	< 0.001
Neither satisfied nor dissatisfied	120(40.3%)	153(51.0%)	
Mostly satisfied	125(41.9%)	60(20.0%)	
Health Status			
Mostly bad	38(6.35%)	43(7.19%)	0.794
Neither good nor bad	100(16.72%)	103(17.22%)	
Mostly good	160(26.76%)	154(25.75%)	
Sleep quality			
Mostly bad	79(26.5%)	104(34.7%)	0.002
Neither good nor bad	105(35.2%)	121(40.3%)	
Mostly good	114(38.3%)	75(25.0%)	
PIL, median (IQR)	93(80–105)	93(79.5–108)	0.622
Resilience, median (IQR)	71(63–82)	67(56–76)	< 0.001

Statistical analysis method: Mann-Whitney U test was performed for continuous variables, Chi-square and Fisher exact tests were performed for categorical variables as appropriate

**Fig. 1:**
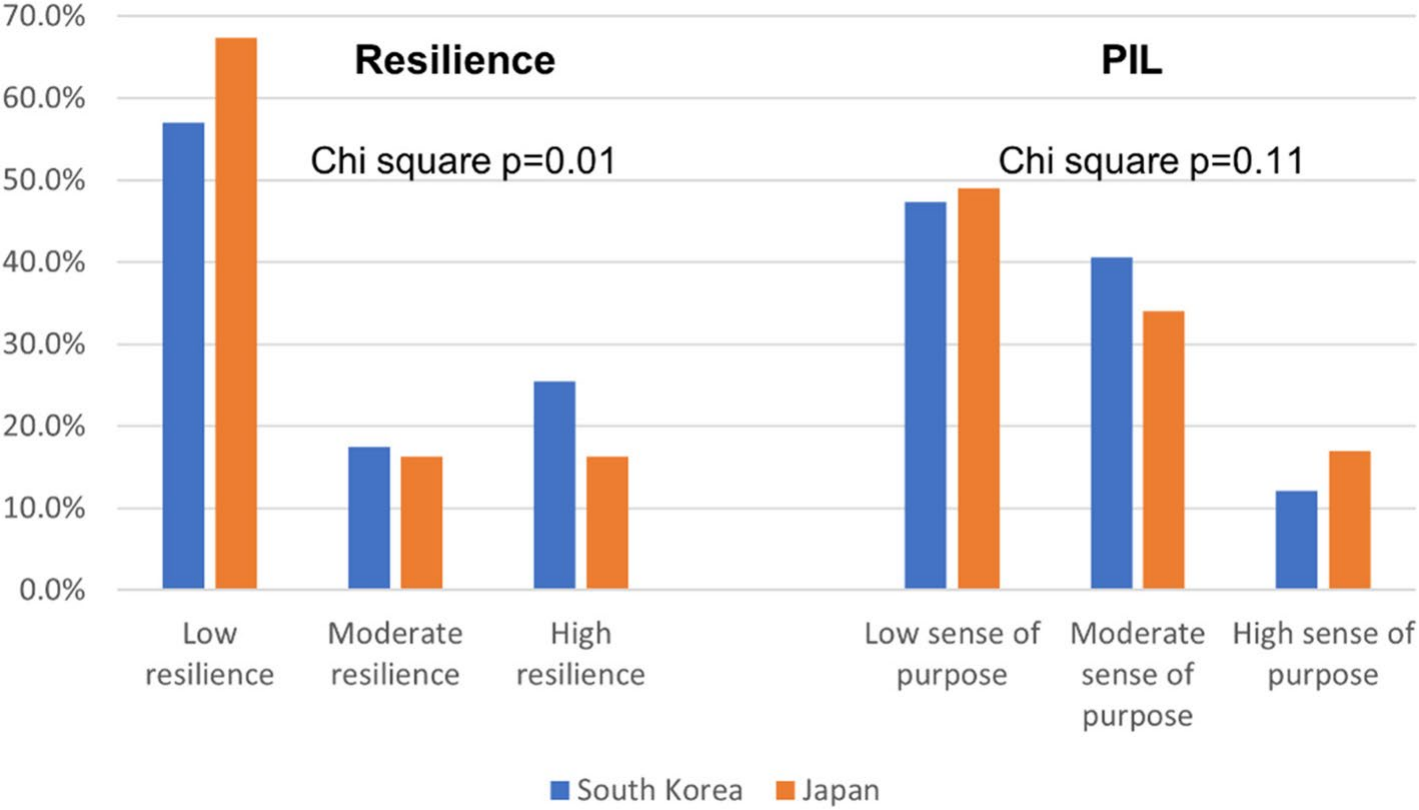
Resilience and PIL levels among South Korean and Japanese university students

There was a significant difference between the resilience levels of the two study populations. Low resilience was found in 57.1% of South Korean and 67.3% of Japanese students; Moderate resilience in 17.5% of South Korean and 16.3% of Japanese students; high resilience in 25.5% of South Korean and 16.3% of Japanese students. PIL was, However not significantly different between students of the two countries with a low sense of purpose in 47.3% of South Korean and 49.0% of Japanese students; a moderate sense of purpose in 40.6% of South Korean and 34.0% of Japanese students; high sense of purpose in 12.1% of South Korean and 17.0% of Japanese students (Fig. 1).

Univariate quantile regression was performed to see the independent association of various factors with resilience level. Among Korean respondents, PIL had a consistent positive association with resilience at all three quantiles. Similarly, participants’ age, knowledge of their own heritage/culture, academic satisfaction, and health status were found to be significantly associated with resilience at all three quantiles. However, affiliation with club/society had a significant positive association at 25th quantile only, physical activity at the median quantile, and financial satisfaction at 75th quantile only, as shown in Table 2.

**Table 2:** Unadjusted association of correlates with resilience levels among South Korean respondents

Variable	Quantile 25 β(95%CI)	Quantile 50 β(95%CI)	Quantile 75 β(95%CI)
PIL	0.66***(0.60; 0.72)	0.6***(0.54; 0.65)	0.57***(0.50; 0.65)
Female gender	−2.00(−7.01; 3.01)	−2.00(−6.33; 2.33)	−2.00(−6.13; 2.13)
(Male = 1)			
Age	1.33*(0.19; 2.46)	2.42***(1.44; 3.41)	1.66***(0.02; 2.31)
Religion			
(No religion = 1)			
Buddhists	4.00(−2.91; 10.91)	1.00(−8.68; 10.68)	−2.00(−9.79; 5.79)
Christians	4.00(−0.15; 8.15)	2.00(−4.75; 6.75)	2.00(−2.69; 6.69)
Knowledge of own heritage			
(Low = 1)			
Moderate	4.00(−0.88; 8.88)	5.00*(0.16; 9.83)	5.00*(0.06; 9.93)
High	7.00**(2.17; 11.82)	8.00** (2.55; 13.44)	9.00**(3.20; 14.79)
Physical activity			
(None = 1)			
1–2 times	1.00(−3.00; 5.00)	2.00(−2.72; 6.72)	2.00(−2.16; 6.16)
> 3 times	5.00(−5.57; 15.57)	8.00**(2.04; 13.95)	2.00(−3.99; 7.99)
BMI			
(Skinny = 1)			
Normal	3.00(−2.04; 8.04)	0(−5.31; 5.31)	4.00(−1.04; 9.04)
Overweight	0 (−7.50; 7.50)	−1.00(−8.39; 6.39)	1.00(−5.98; 7.98)
Obese	−3.00(−9.75; 3.75)	2.00(−13.64;17.64)	0(−12.11;12.11)
Affiliated with Club/society	4.00*(0.19; 7.80)	3.00(−1.12; 7.12)	3.00(−1.48; 7.48)
(Not affiliated = 1)			
Volunteers	4.00(−3.54; 11.54)	−3.00(−12.97; 6.97)	−2.00(−8.05; 4.05)
(Non-volunteers = 1)			
Living arrangement			
(Living alone = 1)			
With family	−7.00(−14.16; 0.16)	−4.00(−9.35; 1.35)	0(−4.13; 4.13)
Dormitory	−2.00(−10.10; 6.10)	−2.00(−8.25;4.25)	0(−5.24; 5.24)
Academic satisfaction			
(Mostly dissatisfied = 1)			
Neither satisfied nor dissatisfied	3.00(−4.03; 10.03)	6.00**(1.79; 10.20)	12.00**(2.98; 21.01)
Mostly satisfied	13.00***(7.78; 19.21)	17.00***(12.61; 21.38)	21.00***(12.53; 29.46)
Financial Satisfaction			
(Mostly dissatisfied = 1)			
Neither satisfied nor dissatisfied	−1.00(−6.58; 4.58)	−4.00(−10.64; 2.64)	−5.00(−10.88; 0.88)
Mostly satisfied	5.00(−0.34; 10.34)	6.00(−1.04; 13.04)	5.00*(0.14; 9.85)
Health Status			
(Mostly bad = 1)			
Neither good nor bad	6.00(−4.18; 16.18)	5.00(−3.14; 13.14)	5.00(−1.53; 11.53)
Mostly good	13.00**(3.02; 22.97)	12.00**(3.86; 20.13)	11.00***(6.26; 15.73)
Sleep quality			
(Mostly bad = 1)			
Neither good nor bad	3.00(−2.56; 8.56)	0(−4.94; 4.94)	0(−5.66; 5.66)
Mostly good	4.00(−1.31; 9.31)	2.00(−3.87; 7.87)	5.00*(0.28; 9.71)

*Significant at *p* 0.05

**Significant at *p* 0.01

***Significant at *p* 0.001

Univariate analysis among Japanese respondents also revealed a consistent significant association of PIL with resilience at all three quantiles. Academic satisfaction, financial satisfaction, health status, and sleep quality were also significantly associated with resilience at all quantiles of resilience, whereas physical activity was significantly associated at 25th and 75th quantiles only, as shown in Table 3.

**Table 3:** Unadjusted association of resilience with correlates among Japanese respondents

Variable	Quantile 25 β(95%CI)	Quantile 50 β(95%CI)	Quantile 75 β(95%CI)
PIL	0.62***(0.55 to 0.69)	0.62***(0.57 to 0.67)	0.54***(0.48 to 0.61)
Female gender	-1.00(-5.05 to 3.05)	0(-4.77 to 4.77)	-3.00(-7.88 to 1.88)
(Male=1)			
Age	0(-2.00 to 2.00)	0(-2.22 to 2.22)	0.5(-1.49 to 2.49)
Religion			
(No religion=1)			
Buddhists	-1.00(-7.80 to 5.80)	-3.00(-11.55 to 5.55)	-2.00(-10.15 to 6.15)
Christians	5.00(-9.33 to 19.33)	11.00(-7.03 to 29.03)	7.00(-10.17 to 24.17)
Shinto	8.00(-3.77 to 19.77)	1.00(-13.80 to 15.80)	-1.00(-15.10 to 13.10)
Knowledge of own heritage			
(Low=1)			
Moderate	2.00(-3.09 to 7.09)	2.00(-3.69 to 7.69)	3.00(-3.69 to 7.69)
High	6.00(-5.30 to 17.30)	4.00(-2.50 to 10.50)	-1.00(-2.50 to 10.50)
Physical activity			
(None=1)			
1-2 times	4.00(-0.11 to 8.11)	4.00(-1.73 to 9.73)	7.00**(1.97 to 12.02)
>3 times	11.00***(5.22 to 16.77)	6.00(-1.68 to13.68)	10.00**(3.10 to16.89)
BMI			
(Skinny=1)			
Normal	2.00(-6.84 to 10.84)	-1.00(-6.84 to 4.84)	-2.00(-7.84 to 3.84)
Overweight/Obese	4.00(-14.07 to 22.07)	-4.00(-13.92 to5.92)	-5.00(-22.78 to 12.78)
Affiliated with Club/society	0(-4.39 to 4.39)	-1.00(-6.27 to 4.27)	0(-4.89 to 4.89)
(Not affiliated=1)			
Volunteers	0(-4.71 to 4.71)	1.00(-4.26 to 6.29)	3.00(-2.06 to8.06)
(Non-volunteers=1)			
Living with family	1.00(-4.62 to 6.62)	0(-5.67 to 5.67)	1.00(-4.53 to 6.53)
(Living alone=1)			
Academic satisfaction			
(Mostly dissatisfied=1)			
Neither satisfied nor dissatisfied	10*(2.09 to 17.90)	6.00(-1.98 to 13.98)	3.00(-2.88 to 8.88)
Mostly satisfied	15***(6.86 to 23.13)	16.00***(8.50 to 23.49)	12.00***(5.80 to 18.19)
Financial Satisfaction			
(Mostly dissatisfied=1)			
Neither satisfied nor dissatisfied	6.00*(0.30 to 11.69)	6.00*(0.69 to 11.30)	4.00(-1.23 to 9.23)
Mostly satisfied	6.00(-2.90 to 14.90)	9.00**(3.12 to 14.87)	8.00**(2.71 to 13.28)
Health Status			
(Mostly bad=1)	8.00(-1.04 to 17.04)	4.00(-3.15 to 11.15)	3.00(-4.68 to 10.68)
Neither good nor bad			
Mostly good	15.00**(5.79 to 24.20)	15.00***(8.80 to 21.19)	13.00**(5.11 to 20.88)
Sleep quality			
(Mostly bad=1)			
Neither good nor bad	6.00**(1.38 to 10.61	5.00(-0.94 to 10.94)	3.00(-2.86 to 8.86)
Mostly good	10.00**(3.44 to 16.55)	11.00**(4.38 to 17.61)	8.00**(1.94 to 14.05)

*Significant at *p* 0.05

**Significant at *p* 0.01

***Significant at *p* 0.001

In the adjusted multivariate model for Korean respondents, purpose in life (PIL) was positively associated with resilience across all quantiles: 25th (β = 0.62, 95% CI: 0.55 to 0.70), 50th (β = 0.56, 95% CI: 0.50 to 0.61), and 75th (β = 0.53, 95% CI: 0.47 to 0.59), indicating a consistent effect throughout the resilience distribution. Club affiliation was associated with higher resilience at the 25th (β = 2.51, 95% CI: 0.34 to 4.68) and 75th quantiles (β = 1.92, 95% CI: 0.01 to 3.84, while female gender was significantly associated with greater resilience only at the median quantile (50th: β = 1.98, 95% CI: 0.11 to 3.85). Participants who were neither satisfied nor dissatisfied with their financial situation reported lower resilience at the 75th quantile (β = − 5.06, 95% CI: − 8.46 to − 1.66) compared to those mostly dissatisfied.

Among the Japanese respondents, a similarly strong positive association was observed between PIL and resilience at all three quantiles: 25th (β = 0.60, 95% CI: 0.53 to 0.67), 50th (β = 0.65, 95% CI: 0.60 to 0.70), and 75th (β = 0.56, 95% CI: 0.48 to 0.63). Financial satisfaction was significantly associated with resilience at the lower (25th) and median (50th) quantiles. Compared to those mostly dissatisfied financially, students who were neither satisfied nor dissatisfied had lower resilience at the 25th (β = − 3.44, 95% CI: − 6.44 to − 0.44) and 50th quantiles (β = − 2.52, 95% CI: − 4.56 to − 0.47). Those who were mostly satisfied also had lower resilience at same quantiles: 25th (β = − 4.76, 95% CI: − 8.44 to − 1.09) and 50th (β = − 2.74, 95% CI: − 5.30 to − 0.18). Additionally, at the 25th quantile, students who were neither satisfied nor dissatisfied with their academic situation exhibited higher resilience compared to those mostly dissatisfied (β = 4.19, 95% CI: 0.38 to 7.99) as shown in Table 4.

**Table 4:** Adjusted association of correlates with resilience levels

	Variable	Quantile 25 β(95%CI)	Quantile 50 β(95%CI)	Quantile75 β(95%CI)
Model 1 Korea	PIL	0.62***(0.55 to 0.70)	0.56***(0.50 to 0.61)	0.53***(0.47 to 0.59)
	Affiliated with club/society	2.51*(0.34 to 4.68)	1.35 (-0.28 to 2.99)	1.92*(0.01 to 3.84)
	(Not affiliated=1)			
	Age	0.21(-0.30 to 0.73)	0.52*(0.06 to 0.97)	0.73*(0.17 to 1.28)
	Female gender (male=1)	0.74 (-1.60 to 3.08)	1.98*(0.11 to 3.85)	2.18(-0.09 to 4.46)
	Financial Satisfaction			
	(Mostly dissatisfied=1)			
	Neither satisfied nor dissatisfied	-1.07 (-6.04 to 3.88)	-1.65(-4.29 to 0.97)	-5.06*( -8.46 to -1.66)
	Mostly satisfied	1.02(-4.06 to 6.11)	1.07(-1.49 to 3.65)	-2.80(-6.43 to 0.83)
Model 2 Japan	PIL	0.60***(0.53 to 0.67)	0.65***(0.60 to 0.70)	0.56***(0.48 to 0.63)
	Financial Satisfaction			
	(Mostly dissatisfied=1)			
	Neither satisfied nor dissatisfied	-3.44*(-6.44 to 0.44)	-2.52*(-4.56 to -4.47)	0.18(-2.49 to 2.85)
	Mostly satisfied	-4.76*(-8.44 to -1.09)	-2.74*(-5.30 to -0.18)	0.48(-3.04 to 4.01)
	Academic satisfaction			
	(Mostly dissatisfied=1)			
	Neither satisfied nor dissatisfied	4.19*(0.38 to 7.99)	2.03(-1.67 to 5.73)	-1.51(-5.98 to 2.96)
	Mostly satisfied	2.88(-1.08 to 6.85)	0.65(-3.15 to 4.45)	-1.82(-6.56 to 2.90)

Variables entered in each model: PIL, age, gender, academic major, academic satisfaction, financial satisfaction, affiliation with club/society, physical activity, sleep quality, and knowledge of own history/culture

Only variables with significant associations are included in the table

*Significant at *p* 0.05

** Significant at *p* 0.01

***Significant at *p* 0.001

## Discussion

One main objective of our study was to assess the level of resilience among university students in South Korea and Japan. We found that more than half of the study participants in either country have low resilience (South Korean 57.1%; Japanese 67.3%) (Fig. 1). South Korean university students had significantly better resilience than their Japanese counterparts, while PIL was not significantly different between the two study groups. Previous studies have found that ethnicity and culture affect the resilience of youth [22, 36]. Although the instruments used to measure the resilience in different studies vary and therefore making the comparison with results of other studies is difficult, nevertheless, the proportion of students with low resilience in our study is substantial, suggesting an overall low resilience of students of this region as compared to university students in other parts of the world [22, 36, 37].

The present study found a robust, positive association between spiritual health (measured by purpose in life) and resilience across all included quantiles of the resilience distribution in both Korean and Japanese students (Table 4). This consistency suggests that a higher sense of purpose benefits students regardless of whether they exhibit low, moderate, or high levels of resilience. Therefore, along with other desirable consequences of better spiritual health, such as a higher life quality [38] and optimism [39], better resilience may also be one of its favorable outcomes. Students with a stronger sense of purpose are more likely to adapt effectively to stress and maintain psychological well-being in the face of adversity. A well-defined sense of purpose not only provides direction but also serves as a psychological anchor that fosters emotional stability during challenging periods. It allows individuals to interpret setbacks as meaningful components of a larger life narrative rather than isolated failures, thereby promoting a sense of coherence and enhancing motivation and perseverance. This orientation may also encourage engagement in proactive coping strategies, health-promoting behaviors, and goal-directed action, all of which contribute to greater psychological resilience. Resilience may partially mediate the observed association between spiritual health and improved mental health outcomes. By enabling individuals to regulate emotions and respond adaptively to stress, resilience translates the abstract concept of purpose into tangible psychological resources. This finding is consistent with previous studies, that reported an association between purpose in life and resilience in the older population of Spain and the United States [40, 41]. However, Johnson EL did not find an association of purpose in life with psychological resilience in his study conducted on college students from the United States [37]. It is important to note that many previous studies that found associations of resilience with spiritual health used spiritual health instruments that included the aspect of religion/connectedness with God [42–45]. Although some studies have shown the influence of religiosity on resilience [46], we did not find a significant association of resilience with religion per se among the youth of South Korea and Japan (Tables 2 and 3). Thus, in the current study, an association of spiritual health and resilience in a population that predominantly follows no religion (South Korean 64.4% Japanese 85.0%) stands as a noteworthy finding, emphasizing the importance of spiritual health in the absence of reliance on faith. It is, therefore, essential to include the spiritual domain in health promotion efforts and encourage initiatives that support a purpose-driven life.

Another finding of our study is the association of resilience with involvement in club activities, with significantly higher resilience levels in students who were affiliated with any type of club or society in South Korea (Table 4). This result is consistent with previous studies where participation in club activities is found to be associated with resilience [47, 48]. The connection between the two factors can be explained by the fact that association with activity clubs in a non-academic setting provides an opportunity to enhance social skills and create a sense of community. In particular, activities like games and music allow students to handle pressure and deal with wins and losses constructively. This finding can serve as a catalyst in designing health promotion strategies that encourage extracurricular engagement as a pathway to improving students’ mental well-being. However, we did not observe a similar association among Japanese participants. One possible explanation may lie in cultural or structural differences in club engagement between the two countries. For instance, the amount of time students spend in club activities can vary substantially by educational system and social expectations, potentially influencing their impact on resilience. Additionally, we lacked information about the quality, intensity, and content of club activities across both contexts, which may further explain this divergence. Future studies should explore these contextual differences in more detail to confirm and better understand the role of club affiliation in fostering resilience among Japanese students.

In the current study, female Korean students demonstrated significantly higher resilience at the median quantile, while no significant gender differences were observed among Japanese students. Similarly, age was positively associated with resilience among Korean students at the median and upper quantiles, but not among their Japanese counterparts. These findings suggest that both gender and age may play a more prominent role in resilience within the Korean context. The literature on gender differences in resilience remains inconclusive, with some studies reporting higher resilience in males, others in females, and many showing no significant difference [49–51]. Such inconsistencies may be explained by varied cultural expectations, social roles, and differences in coping styles across populations. Regarding age, prior research indicates that resilience tends to increase over time as individuals accumulate life experience and develop more mature coping strategies. The lack of association in the Japanese sample may be explained by their comparatively younger age profile (median age 19 vs. 21 in Korean students), which may have limited the observable variation in resilience. These findings highlight the importance of considering cultural and developmental contexts when examining demographic influences on resilience and underscore the need for age- and gender-sensitive mental health support strategies in university settings.

Academic satisfaction emerged as a significant correlate of resilience among Japanese students, particularly at the lower end of the resilience distribution. This finding is also consistent with some previous studies that have identified the association of better academic performance with resilience [23, 52]. The authors of these studies have justified this association by arguing that better academic performance and overall academic satisfaction increase self-efficacy and self-confidence, thereby enhancing resilience and coping abilities. Previous studies have also indicated the contribution of academic satisfaction judgment to subjective well-being by enhancing positive emotions [53]. However, the relationship may be bidirectional. It is plausible that students with greater resilience are more capable of managing academic challenges, thereby perceiving their academic experience more positively. The use of quantile regression in this study offers additional nuance by highlighting that the association may be more pronounced among those with lower baseline resilience. Notably, this relationship was not observed among South Korean students, which may point to contextual differences in educational environments, academic pressure, or perceptions of academic success.

An unexpected pattern emerged regarding the association between financial satisfaction and resilience. In South Korea, students who were neutral about their financial situation reported lower resilience at higher levels of the resilience distribution, while in Japan, both neutral and mostly satisfied students showed lower resilience at lower to mid-levels compared to those who were mostly dissatisfied. These findings contrast with previous research, which has generally shown a positive association between higher socioeconomic status and greater resilience [54, 55]. The observed relationships in our study suggest a more complex and potentially context-dependent dynamic. It is possible that subjective financial satisfaction does not always reflect actual financial stability, and that individuals who report dissatisfaction may simultaneously develop stronger coping mechanisms or receive more external support. Financial security often serves as a buffer against stress and provides resources for coping—such as access to self-care and stress-relief activities—which can enhance resilience. However, resilience is shaped by a combination of other factors, including coping strategies, personality traits, and social support systems. Therefore, some individuals may maintain higher resilience even amid financial dissatisfaction, while others with greater financial satisfaction may not necessarily experience higher resilience. Given the unexpected association observed between financial satisfaction and resilience, future studies should explore potential mediating factors - such as employment status, parental or social support, and coping strategies - that may influence this relationship. Mediation analysis could help clarify the underlying mechanisms and provide a more nuanced understanding of how subjective socioeconomic status affects resilience in different student populations.

The association of resilience with physical activity and BMI has previously been reported in some studies [56–58]. However, in the current study, physical activity or BMI is not found to be significantly associated with resilience in the final model, although we did find a positive association of resilience with physical activity in both countries in the unadjusted analysis (Tables 2 and 3). In the present study, knowledge of own heritage and culture was associated with resilience in unadjusted analysis among South Korean students. However, the association was rendered insignificant after the inclusion of other variables in the fully adjusted model. While there is no conclusive evidence on the association of this characteristic with resilience, studies from some cultures report good knowledge of own culture and history as a correlate of higher resilience in the Indigenous populations [59, 60]. We suggest it as an area for further research to substantiate the importance of such knowledge in terms of bolstering resilience and psychological well-being.

Between-country comparisons (Table 1) revealed that South Korean students were more actively involved in volunteer activities and activity clubs compared to their Japanese counterparts. These forms of engagement have been linked to better psychological well-being and self-rated health [61, 62]. They also reported greater knowledge of their own history and culture, which may contribute to a stronger sense of identity. In contrast, Japanese students reported lower financial satisfaction and poorer sleep quality, both of which are associated with reduced resilience. These differences, alongside broader cultural factors, may help explain the higher resilience levels observed among South Korean university students.

Our study has several strengths. The survey was conducted in local languages using validated instruments, ensuring clarity and measurement reliability. The synchronized survey period across both countries minimized temporal bias. Covariates were carefully selected through literature review and team discussions to reduce confounding. Additionally, the use of quantile regression enabled robust analysis across the resilience distribution, offering deeper insights beyond mean-based approaches. The findings of this study should be interpreted in light of several limitations. First, the reliance on self-reported data may have introduced response and information biases. Second, a non-probability sampling approach was employed, as is typical in web-based research, resulting in a self-selected volunteer sample that may be subject to non-sampling bias. While participants were recruited from three universities in South Korea and one in Japan, which is the country’s oldest private university, known for its diverse student population, the limited number of institutions and geographic locations included restricts the generalizability of the results, as the sample may not fully capture the heterogeneity of the broader student populations in either country. Differences in institutional cultures and regional socioeconomic factors could influence the observed relationships, limiting the extent to which these findings can be extrapolated to other university settings. Future research should include a more diverse range of universities to improve external validity. Finally, while the Purpose in Life (PIL) scale effectively captures a key aspect of spiritual health related to meaning and purpose, it does not encompass the broader, multidimensional nature of spirituality, which may limit the interpretation of our findings in this domain.

Given the generally low levels of resilience observed among university students in both South Korea and Japan, there is a clear need to prioritize interventions that enhance students’ internal coping resources. This study provides several actionable insights for student support services and mental health initiatives in higher education. The consistently positive association between purpose in life (PIL) and resilience underscores the potential of purpose-driven interventions - such as reflective workshops, goal-setting coaching, and curricular activities focused on values exploration - to foster psychological resilience. The positive impact of club affiliation at both the lower and upper ends of the resilience spectrum highlights the importance of promoting meaningful peer connections and extracurricular involvement, particularly for students experiencing either heightened vulnerability or strong adaptation. Additionally, the association between academic satisfaction and resilience at lower quantiles suggests that proactive academic advising and supportive learning environments can be critical in supporting students with lower resilience. Gender-specific finding points to the need for resilience-building strategies that are sensitive to individual and contextual variations, rather than relying solely on broad demographic categorizations. We recommend incorporating tailored resilience-enhancing programs into university curricula. Evidence-based interventions, such as the Penn Resilience Program and Mindfulness-Based Stress Reduction (MBSR) [63, 64], can be adapted to address the social, psychological, and demographic factors identified in this study. Furthermore, the findings may inform the development of survey instruments designed to identify students at risk of mental health difficulties due to low resilience and coping capacity, enabling early, non-stigmatizing intervention. Future research should employ qualitative and longitudinal designs to deepen understanding of these associations and guide the refinement of targeted resilience-building strategies.

## Conclusion

Given the persistently high rates of psychological stress, mental health challenges, and suicidal tendencies among youth in South Korea and Japan, it is imperative to focus on cultivating the inner strengths of this population to better equip them for future adversities. The substantial prevalence of low resilience among university students in both countries underscores the urgent need to implement resilience-enhancing interventions. Our study identified several factors associated with greater resilience, including a strong sense of purpose in life, academic satisfaction, club participation, and demographic characteristics such as age and gender. These elements can inform health promotion strategies aimed at improving student well-being. To deepen understanding and guide more effective interventions, further qualitative and longitudinal research is warranted to explore the complex nature of resilience and its contributing influences in this demographic.

## Supplementary Information

Supplementary Material 1.

## Data Availability

Data are available from the authors upon reasonable request.

## Abbreviations

PIL: Purpose in life scale
RS14: 14-item resilience scale
BMI: Body mass index
IQR: Interquartile range
β: Coefficient
CI: Confidence intervals
