# Supplementary material for: Evaluating resilience levels and their association with spiritual health and other factors among university students from South Korea and Japan; a comparative cross-sectional study
Source: BMC Public Health. 2025 Oct 14;25:3490. doi: 10.1186/s12889-025-24738-1 (PMC12522988; doi:10.1186/s12889-025-24738-1)
Supplement: Supplementary file 1 — Supplementary Material 1. [file 12889_2025_24738_MOESM1_ESM.docx]

**Supplementary Material**

**Supplementary Table 1** Survey questions on covariates

| Variable | Question Asked | Response Options |
| --- | --- | --- |
| Gender | What is your gender? | Male/Female/Other |
| Age | What is your year of birth? (write in example: 1994) | Open response |
| Religion | Do you have a religious affiliation? | Buddhism/Shinto/Christianity/Islam/Taoism/No religion/Others |
| Field of study | What is your current field of study? | Law or politics/Economics or Management or commerce/ Society or media/international relations/Literature or humanities or psychology/foreign language/education/home economics or life/Art/health or sports/Liberal arts/science/Engineering/agriculture or animals or fisheries/medical or dental/nursing or hygiene/other |
| Knowledge of own history and culture | How much do you know about your roots (history and culture)? | Nothing at all/A little/Some/Quite a lot/A great deal |
| Physically activity | Over the last 7 days have you exercised or played sports or games that made you sweat and breathe hard (e.g. basketball, netball, football, riding a bike, running) | No/1–2 times/ 3 or more times |
| Affiliated with Club/society | Do you belong to any club/organization/society | Yes/No  Mention the name |
| Volunteering experience | Do you do or have you ever done volunteer work? | Yes/No |
| Living arrangement | Please tell me about your living arrangements. | Living alone/living with family/Dormitory/other |
| Academic satisfaction | Are you satisfied with your studies and college life? Please choose the one that is closest to your current status. | Very satisfied/Somewhat satisfied/Neither satisfied nor dissatisfied/Somewhat dissatisfied/Very dissatisfied |
| Financial Satisfaction | Are you currently financially satisfied? Please choose the one that is closest to your current status. | Very satisfied/Somewhat satisfied/Neither satisfied nor dissatisfied/Somewhat dissatisfied/Very dissatisfied |
| Health Status | How would you rate your health? | Very good/Good/Neutral/Not very good/poor |
| Sleep quality | How was your sleep quality in the last 4 weeks? | Very good/Good/Neutral/Not very good/Poor |
| BMI | What is your height in centimeters?  What is your weight in kilograms? | Open responses |
